# Supplementary material for: D-serine mitigates cell loss associated with temporal lobe epilepsy
Source: Nat Commun. 2020 Oct 2;11:4966. doi: 10.1038/s41467-020-18757-2 (PMC7532172; doi:10.1038/s41467-020-18757-2)
Supplement: Supplementary file 1 — Supplementary Information [file 41467_2020_18757_MOESM1_ESM.pdf]

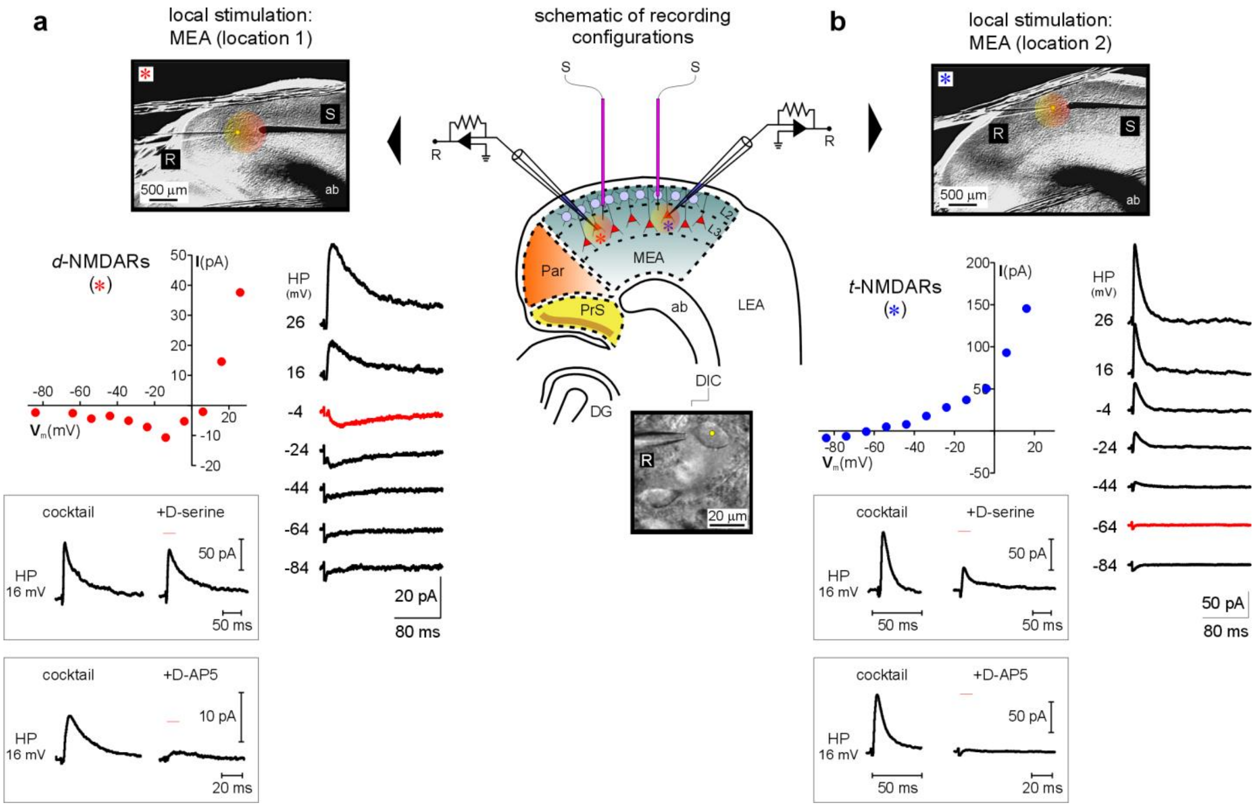

Supplementary Fig 1: **Expression and electrophysiological properties of *t*- and *d*-NMDARs in the MEA. a**-**b**, Experimental paradigm and schematic of model (*center*) used for studying electrophysiological properties of NMDAR-mediated excitatory post-synaptic currents (EPSCs) evoked in distinct populations of layer 3 pyramidal neurons (*bottom inset*: high-powered image of a recorded neuron under IR-DIC optics) in the medial (**a**, location 1, *left panel*) and lateral (**b**, location 2, *right panel*) portions of the MEA during minimal stimulation (**S**, stimulating electrode) of local intracortical afferents; Par, parasubiculum; PrS, presubiculum; ab, angular bundle; LEA, lateral entorhinal area; DG, dentate gyrus of the hippocampus (Hip). Recording electrodes (**R**, 1.2-2 μm tip diameters; 3-6 MΩ) contained the following (in mM): 120 cesium gluconate, 1 $MgCl_2$, 1 $CaCl_2$, 11 CsCl, 10 HEPES, 2 NaATP, 0.3 NaGTP, 1 QX-314, 11 EGTA, and 20 biocytin (pH 7.3 was corrected with Cs-OH, 290 mOsm). Note differences between the current-voltage (I-V) relationships of NMDAR-mediated EPSCs (isolated pharmacologically using a cocktail of 50 μM picrotoxin and 10 μM NBQX in aCSF) in neurons from location 1 that express *d*-NMDARs with conventional I-Vs (*red*) and those from location 2 that express *t*-NMDARs with outwardly-rectifying I-Vs (*blue*). Traces (averaged from 5 or more consecutive records) corresponding to holding potentials (HP) at which the EPSCs reverse polarity are indicated in red. Boxed insets showcase the effects of D-serine (10 μM, *top*) and D-AP5 (40 μM, *bottom*) on EPSC amplitude for the two receptor types at the indicated holding potential.

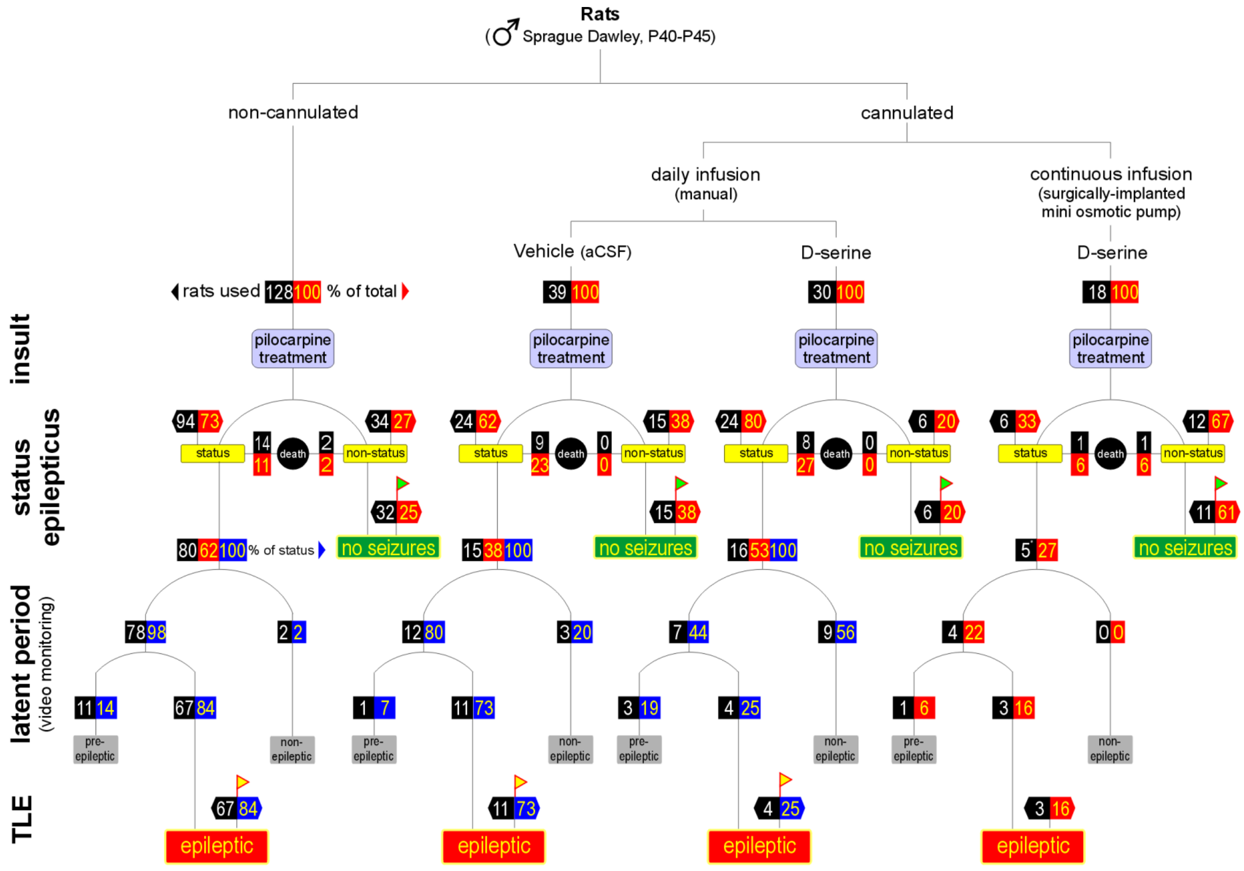

Supplementary Fig 2: **Animals used in our behavioral assay of D-serine on TLE.** Tree diagram showing behaviorally observed outcomes (status epilepticus, frank seizures, epilepsy or death) of pilocarpine-treatment (insult) under conditions identified in **a**. The boxed numbers indicate total animals (*black*), % of total animals (*red*) or % of animals with status epilepticus (status, *blue*), under various regimens (changes are flagged to encourage comparison). All video recordings of behavior are available upon request.

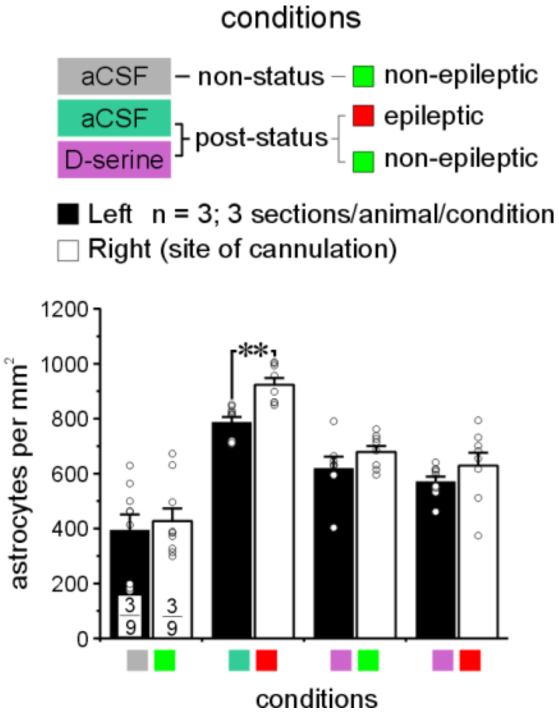

Supplementary Fig 3: **Astrocyte densities in left and right hemispheres of control, epileptic and D-serine treated animals.** Raw data and histogram of astrocyte densities **(**immunoassayed with GFAP) in layer 3 of MEA in animals under the conditions indicated (color codes show segregation of cohorts based on treatment regimen and final outcomes). Data within bar plots indicates number of animals used (*numerator*) and the total number of sections assayed for each condition (*denominator)*. Error bars represent SEM. ** $p < 0.01$, Student's t-test.

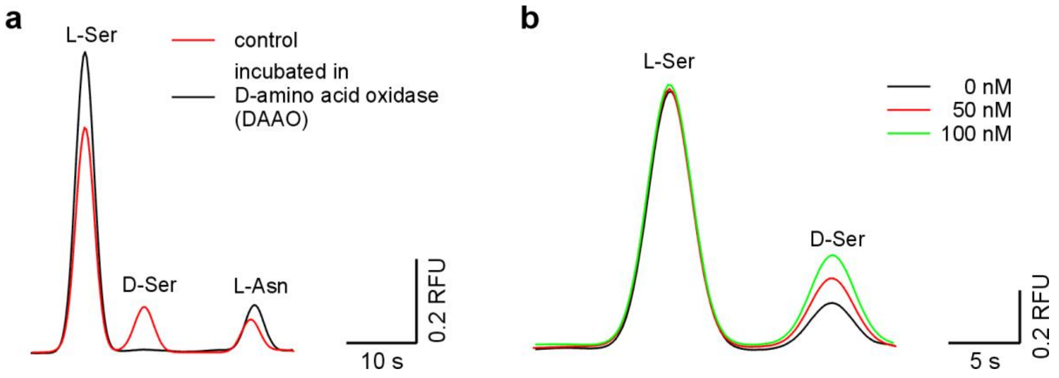

Supplementary Fig 4: **Validation of D-serine measurements using MEKC. a,** Overlay of normalized electropherograms of a tissue sample from MEA analyzed before (*red*) and after (black) incubation with D-amino acid oxidase (DAAO), flavin adenine dinucleotide (FAD), and catalase in a 37°C water bath for 1 hour. Note the absence of the D-Ser peak following incubation with DAAO. **b,** Overlay of normalized electropherograms of a tissue sample from MEA spiked with varying concentrations of the D-Ser standard indicated. Note the specificity of the effect and the elevation in D-ser peak proportionate with increasing concentrations of added D-serine.

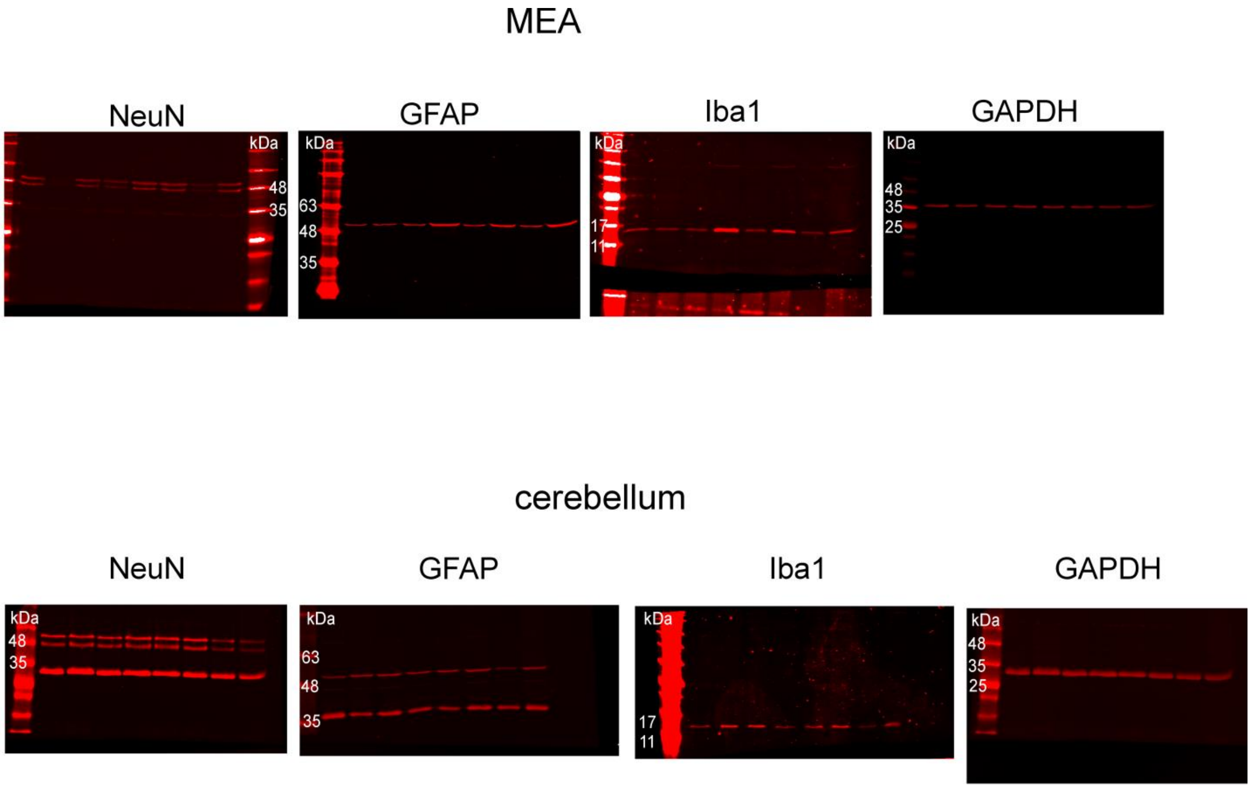

Supplementary Fig 5: Un-cropped versions of immunoblots shown in Fig. 4d for NeuN (*neurons*), GFAP (*astrocytes*) and Iba1 (*microglia*) for MEA (*top panel*) and cerebellum (*bottom panel*) harvested from non-status and post-status rats 1, 5, 12 and 29-days post-insult. GAPDH was used as loading control. The following antibodies were used for immunoblotting: anti-NeuN (rabbit, Millipore, MABN140); anti-GFAP (rabbit, Abcam, ab206586); anti-IbaI (rabbit, Wako, 016-20001); and anti-GAPDH (rabbit, Sigma, G9545).
