# Supplementary material for: D-serine mitigates cell loss associated with temporal lobe epilepsy
Source: Nat Commun. 2020 Oct 2;11:4966. doi: 10.1038/s41467-020-18757-2 (PMC7532172; doi:10.1038/s41467-020-18757-2)
Supplement: Supplementary file 3 — Reporting Summary [file 41467_2020_18757_MOESM3_ESM.pdf]

nature research

Corresponding author(s): Sanjay Kumar

Last updated by author(s): Sep 4, 2020

Reporting Summary

Nature Research wishes to improve the reproducibility of the work that we publish. This form provides structure for consistency and transparency in reporting. For further information on Nature Research policies, see our Editorial Policies and the Editorial Policy Checklist.

Statistics

For all statistical analyses, confirm that the following items are present in the figure legend, table legend, main text, or Methods section.

n/a	Confirmed	
☐	☒	The exact sample size (n) for each experimental group/condition, given as a discrete number and unit of measurement
☒	☐	A statement on whether measurements were taken from distinct samples or whether the same sample was measured repeatedly
☐	☒	The statistical test(s) used AND whether they are one- or two-sided<br>*Only common tests should be described solely by name; describe more complex techniques in the Methods section.*
☒	☐	A description of all covariates tested
☐	☒	A description of any assumptions or corrections, such as tests of normality and adjustment for multiple comparisons
☐	☒	A full description of the statistical parameters including central tendency (e.g. means) or other basic estimates (e.g. regression coefficient) AND variation (e.g. standard deviation) or associated estimates of uncertainty (e.g. confidence intervals)
☐	☒	For null hypothesis testing, the test statistic (e.g. F, t, r) with confidence intervals, effect sizes, degrees of freedom and P value noted<br>*Give P values as exact values whenever suitable.*
☒	☐	For Bayesian analysis, information on the choice of priors and Markov chain Monte Carlo settings
☒	☐	For hierarchical and complex designs, identification of the appropriate level for tests and full reporting of outcomes
☒	☐	Estimates of effect sizes (e.g. Cohen's d, Pearson's r), indicating how they were calculated

Our web collection on statistics for biologists contains articles on many of the points above.

Software and code

Policy information about availability of computer code

Data collection	Stereo Investigator software (Version 6.0)
Data analysis	Fiji-ImageJ (Version 1.52) ; GraphPad Prism (Version 7)

For manuscripts utilizing custom algorithms or software that are central to the research but not yet described in published literature, software must be made available to editors and reviewers. We strongly encourage code deposition in a community repository (e.g. GitHub). See the Nature Research guidelines for submitting code & software for further information.

Data

Policy information about availability of data

All manuscripts must include a data availability statement. This statement should provide the following information, where applicable:

- Accession codes, unique identifiers, or web links for publicly available datasets
- A list of figures that have associated raw data
- A description of any restrictions on data availability

All figures in the manuscript were made from experimentally obtained raw data, available upon request.

All video recordings documenting seizure behavior have been uploaded to a Microsoft Office 365 SharePoint site created for this purpose by the FSU College of Medicine. Because of the sensitive nature of displaying animal work, we have restricted its viewing to the editors and reviewers of this manuscript. The videos have been organized by groups and navigating through them is self-explanatory. We need a valid e-mail address for us to invite you to the SharePoint site and enable viewing of the videos. This is a secure way in which this content can be made available through our institution.

April 2020

Field-specific reporting

Please select the one below that is the best fit for your research. If you are not sure, read the appropriate sections before making your selection.

- [x] Life sciences
- [ ] Behavioural & social sciences
- [ ] Ecological, evolutionary & environmental sciences

For a reference copy of the document with all sections, see nature.com/documents/nr-reporting-summary-flat.pdf

Life sciences study design

All studies must disclose on these points even when the disclosure is negative.

Sample size	no sample-size calculation was performed. Preliminary studies undertaken indicated strong trends that were either statistically significant or non-significant. We expanded our initial work for rigor and reproducibility by increasing sample size in most of our experiments. Given the in vivo nature of our work and the small SDs precluded the use of power analysis. Our lowest sample size was n = 3 from in-vivo work.
Data exclusions	Data was generally not excluded from the analysis except in rare cases where the experiment could be completed and/or was beyond 1.5 times the inter quartile range (IQR). For example, tissue was lost during processing and this is explicitly acknowledged in the manuscript (figure 2a).
Replication	Experiments were repeated in animals belonging to different cohorts at least on two separate occasions. All attempts at replication were successful.
Randomization	Animals in a cohort were treated similarly during the pilolcarpine procedure and there was no way of knowing which animal in a given cohort would become epileptic. Animals in a cohort were selected randomly post-status to receive either d-serine or aCSF (vehicle).
Blinding	All analysis was done blind.

Reporting for specific materials, systems and methods

We require information from authors about some types of materials, experimental systems and methods used in many studies. Here, indicate whether each material, system or method listed is relevant to your study. If you are not sure if a list item applies to your research, read the appropriate section before selecting a response.

Materials & experimental systems

n/a	Involved in the study	
[ ]	[x]	Antibodies
[x]	[ ]	Eukaryotic cell lines
[x]	[ ]	Palaeontology and archaeology
[ ]	[x]	Animals and other organisms
[x]	[ ]	Human research participants
[x]	[ ]	Clinical data
[x]	[ ]	Dual use research of concern

Methods

n/a	Involved in the study	
[x]	[ ]	ChIP-seq
[x]	[ ]	Flow cytometry
[x]	[ ]	MRI-based neuroimaging

Antibodies

Antibodies used	All antibodies used were obtained commercially and have been used widely in the literature. The following antibodies were utilized in this study (all of these are mentioned explicitly in the methods): anti-NeuN, clone 27-4 (rabbit, Millipore, MABN140, Lot: 3027152); anti-GFAP (rabbit, Abcam, ab206586, Lot: GR3173735-2); anti-Ibal (rabbit, Wako, 016-20001, Lot: PTH4471); and anti-GAPDH (rabbit, Sigma, G9545, Lot: 127M4814V), anti-NeuN Alexa-555, clone A60 (Millipore, mouse; MAB377A5, Lot: 3011825) and anti-GFAP Alexa-488 (Millipore, mouse; MAB3402X, Lot: 3012095) or anti-NeuN, clone 27-4 (Millipore, rabbit; MABN140, Lot: 3027152) and anti-CD11b/c (OX42) (Abcam, mouse; ab1211, Lot: GR3214392-2), anti-serine racemase (GeneTex; GTX83567, 9E8), goat anti-rabbit Alexa-405 (Invitrogen, A-31556, Lot: 1984053) and goat anti-mouse Alexa-488 (Invitrogen; A-11001, Lot: 1787787), goat anti-mouse biotin (Invitrogen, B-2763, Lot: 1901331) and Streptavidin Alexa-594 (Invitrogen, S11227, Lot: 1991448). Dilutions used for each antibody specified in the methods section of the manuscript.
Validation	Validation was provided by the vendor for all antibodies used in this study. A primary negative control was also used to validate the anti-serine racemase antibody.

Animals and other organisms

Policy information about studies involving animals; ARRIVE guidelines recommended for reporting animal research

Laboratory animals	Male Sprague-Dawley rats (P40-P45), commercially obtained from Envigo.

Wild animals	No wild animals were used in the study.
Field-collected samples	No field collected samples were used in this study.
Ethics oversight	All experiments were carried out in accordance with the National Institutes of Health Guide for Care and Use of Laboratory Animals and were approved by the Florida State University Institutional Animal Care Committee.

Note that full information on the approval of the study protocol must also be provided in the manuscript.
